# Global transcriptome analysis of alfalfa reveals six key biological processes of senescent leaves

**DOI:** 10.7717/peerj.8426

**Published:** 2020-01-21

**Authors:** Jianbo Yuan, Xinbo Sun, Tao Guo, Yuehui Chao, Liebao Han

**Affiliations:** 1 College of Grassland Science, Beijing Forestry University, Beijing, China; 2 College of Agronomy, Hebei Agricultural University, Key Laboratory of Crop Growth Regulation of Hebei Province, China

**Keywords:** Leaf senescence, Alfalfa, Differentially expressed genes, Transcriptome

## Abstract

Leaf senescence is a complex organized developmental stage limiting the yield of crop plants, and alfalfa is an important forage crop worldwide. However, our understanding of the molecular mechanism of leaf senescence and its influence on biomass in alfalfa is still limited. In this study, RNA sequencing was utilized to identify differentially expressed genes (DEGs) in young, mature, and senescent leaves, and the functions of key genes related to leaf senescence. A total of 163,511 transcripts and 77,901 unigenes were identified from the transcriptome, and 5,133 unigenes were differentially expressed. KEGG enrichment analyses revealed that ribosome and phenylpropanoid biosynthesis pathways, and starch and sucrose metabolism pathways are involved in leaf development and senescence in alfalfa. GO enrichment analyses exhibited that six clusters of DEGs are involved in leaf morphogenesis, leaf development, leaf formation, regulation of leaf development, leaf senescence and negative regulation of the leaf senescence biological process. The WRKY and NAC families of genes mainly consist of transcription factors that are involved in the leaf senescence process. Our results offer a novel interpretation of the molecular mechanisms of leaf senescence in alfalfa.

## Introduction

Leaves are key photosynthetic organs that produce carbohydrates that provide energy for plant development. In the last stage of leaf development, the photosynthate-producing ability of the leaf is weakened, and the leaf enters the senescence stage (Quirino et al., 2000). During its life span, the leaf undergoes three main developmental phases: initial leaves, maturation, and senescence (Buchanan-Wollaston, 1997). Much more nutrient input is initially needed to support leaf growth and development. The mature leaves are mainly responsible for providing a source of carbon, and this function continues until the onset of senescence. During leaf senescence, the nutrients in leaves, including nitrogen, metals, and phosphorus, are reallocated to other tissues and organs to support plant development. Moreover, the leaf cells undergo significant changes in their cellular metabolism and cellular structures are degenerated (Rattan & Hayflick, 2016; Buchanan-Wollaston, 1997; Zentgraf et al., 2004; Nam, 1997). Leaf senescence is a complex organized developmental process. Initial senescence affects the chloroplasts first and the nucleus and mitochondria last (Cavaiuolo et al., 2015; Avila-Ospina et al., 2015; Quirino, Normanly & Amasino, 1999). Leaf senescence represents a key developmental phase in annual and perennial species, and it is as complex as the other developmental phrases (Buchanan-Wollaston, 1997). Leaf senescence limits the yield of crop plants such as vegetables and fruit. Thus, the study of leaf senescence is important because it will allow us to understand the fundamental biological processes and molecular mechanisms in plants. This knowledge may provide a means to control leaf senescence to improve the traits of crop plants (Lim, Kim & Gil Nam, 2007).

Leaf senescence is a complex molecular regulatory process. Several genes are involved in leaf senescence including *senescence-associated transcriptional repressor 12* (*SAG12*), *plastid ribosomal protein small subunit 17*, and *onset of leaf death1* (Gan & Amasino, 1997; Sade et al., 2017; Jing et al., 2002). Several WRKY transcription factor (TF) genes, such as *AtWRKY6* and *AtWRKY53*, were also found to be involved in leaf senescence. The expression of *AtWRKY6* triggers senescence and is activated by several signal factors. *AtWRKY53* plays a regulatory role in early stages of leaf senescence (Robatzek & Somssich, 2001; Hinderhofer & Zentgraf, 2001). The NAC family of TFs plays an important role in leaf senescence; for example, the function of *AtNAP* mutant can be restored in *Arabidopsis* by overexpression of the *AtNAP* gene. Furthermore, overexpression of wild-type *AtNAP* induces precocious senescence (Guo & Gan, 2006). Additionally, regulated protein degradation plays an important role in the control of leaf senescence; for example, ORE9 limits leaf longevity by removing target proteins that result in the delay of leaf senescence in *Arabidopsis* (Woo et al., 2001). The *delayed-leaf-senescence 1* mutant expresses defective R-transferase protein, which results in delayed leaf senescence (Yoshida et al., 2002). The accumulation of senescence-associated receptor-like kinase regulates leaf senescence in *Phaseolus vulgaris* (Hajouj, Michelis & Gepstein, 2000).

Alfalfa is a legume and an important forage crop worldwide. It is widely used as animal feed. Animals mainly eat the stems and leaves because they are the most nutritious. Leaf senescence may reduce the biomass of alfalfa. A previous study reported that controlling harvesting time is a key factor influencing the biomass of alfalfa (Stavarache et al., 2015). Minimizing nitrogen (N) losses via ammonia (NH_3_) volatilization increases the yield of alfalfa (Li et al., 2018). Mixing alfalfa with grass species can affect the biomass of alfalfa (Maamouri, Louarn & Julier, 2014). Alternate furrow irrigation is a water-saving technique that can increase biomass and improve the quality of alfalfa (Xiao et al., 2015). However, most studies have only reported how to improve the biomass of alfalfa at a mainly physiological level. Our understanding of the molecular mechanism of leaf senescence and its influence on the biomass of alfalfa is still limited.

High-throughput next-generation DNA sequencing (NGS) technologies have allowed RNA analyses to be performed on a massive scale through cDNA sequencing (RNA-seq). NGS technologies are very powerful tools for RNA-seq and genomic studies because they are high throughput, and the read length and error rate can be determined (Metzker, 2010; Ozsolak & Milos, 2011). Several studies have used RNA-seq to investigate leaf senescence in plants such as *Medicago truncatula*, *Arabidopsis*, *Sorghum bicolor*, *Gossypium hirsutum*, *Panicum virgatum*, and maize (Buchanan-Wollaston et al., 2005; De Michele et al., 2009; Li et al., 2010; Lin et al., 2015; Palmer et al., 2015; Wu et al., 2016). Alfalfa is allogamous and autotetraploid that result in genetic background complexity (Gherardi et al., 1998). *Medicago truncatula* is diploid that finished genomic sequencing. It was close relative of alfalfa (Young et al., 2011). In this study, we identified differentially expressed genes (DEGs) in young, mature, and senescent leaves using RNA-seq, and further predicted the functions of key genes related to leaf senescence using bioinformatics analysis methods.

## Material and Methods

### Cultivation of alfalfa

The Baoding cultivar of alfalfa was obtained from the Institute of Animal Science of Chinese Academy of Agricultural Sciences (Beijing, China). Alfalfa is cultivated in Daejeon (Baoding, Hebei province, China). The average annual temperature is 13 °C. Annual average maximum temperature is 18 °C. Annual average minimum temperature is 8 °C. The average annual rainfall is 532 mm. The sampling time is April, 2016. The age of the samples leaves was mature plant. The young, mature, and senescent alfalfa leaves were prepared for RNA-seq. Three biological replicates were performed for each sampling site. T01, T02, and T03 represent samples from the young leaf group; T04, T05, and T06 represent samples from the mature leaf group; and T07, T08, and T09 represent samples from the senescent leaf group. All samples were treated with liquid nitrogen and stored at −80 °C prior to RNA-seq.

### Chlorophyll detection

Chlorophyll was broken down in the leaf senescence development of more mature plants (Süssenbacher et al., 2019). Chlorophyll a and Chlorophyll b contents were measured from leaves of three development stages. 0.08 g of leaves were weighed and ground into a 10mL centrifuge tube. We added 8ml of 95% ethanol, and had it stand still in the dark for 48 h. The absorbance value of the solution was measured using a UV spectrophotometer. The wavelength was 665, 649 and 470nm, and was adjusted with 95% ethanol.

### RNA isolation and quality testing

RNA sequencing was performed on a total of nine samples. T01, T02, and T03 represented three biological replicates in the young leaf group of alfalfa. T04, T05, and T06 represented three biological replicates in the mature leaf group. T05, T06, and T07 represented three biological replicates in the senescent leaf group. Total RNA from each leaf sample was isolated using the TIANGEN RNA Extraction Kit (Beijing, China). The RNase-Free DNase I Set (Omega Bio-Tek, Georgia, USA) was used to remove contaminating genomic DNA. The concentration and purity were checked using the NanoDrop 8000 Spectrophotometer (Thermo Fisher Scientific, Waltham, MA, USA). The RNA integrity was evaluated using the Agilent 2100 Bioanalyzer System (Agilent Technologies Inc., Santa Clara, CA, USA). For RNA-seq.

### Construction of cDNA library and sequencing

A total of 3 µg RNA per sample was used for RNA-seq. The Oligo was enriched with eukaryotic mRNA. The mRNA was broken into fragments using fragmentation buffer. The mRNA was the template, and six random hexamers were used to synthesize the first chain of cDNA. After adding buffer, dNTPs, DNA polymerase I, and RNase H, and the second chain of cDNA were synthesized. The cDNA then underwent end repair, poly (A) tail synthesis, and ligation to sequencing adapters. AMPure XP beads were used to purify the double-stranded cDNA. After the cDNA library was obtained, the quality was checked using the Agilent 2100 Bioanalyzer System. The libraries were sequenced using the Illumina HiSeq X-ten (Life Sciences Company, San Francisco, California, USA).

### Quality control, assembly and functional annotation

After sequencing, the low-quality data were filtered to obtain clean reads. The quality of the reads was evaluated using the Phred value (Q_−score_ = −10 × log_10_P). The qualified data were assembled using Trinity software. Simple sequence repeats (SSRs) of the clean reads were identified using the microsatellite identification tool (MISA). Single nucleotide polymorphisms (SNPs) in clean reads were evaluated by GATK (McKenna et al., 2010). Gene expression was evaluated using fragments per kilobase of transcript per million mapped reads (FPKM), and the expression abundance of unigenes was obtained (Trapnell et al., 2010). Unigenes were screened against the NR and Swiss-Prot databases using BLAST software (Deng et al., 2006; Coordinators, 2016; UniProt Consortium, 2016). The predicted amino acid sequences of the unigenes were screened against the Pfam database using HMMER software to gain annotation information (Nastou et al., 2016; Finn et al., 2013). The functional annotations of the unigenes were screened against the Clusters of Orthologous Groups (COGs; http://www.ncbi.nlm.nih.gov/COG), Gene Ontology (GO; http://www.geneontology.org/), and the Kyoto Encyclopedia of Genes and Genomes (KEGG; http://www.genome.jp/kegg) databases. The BLAST parameter *E*-value was not greater than 1^*e*−5^ and the HMMER parameter *E*-value was not greater than 1^*e*−10^.

### Annotation of differentially expressed genes

Differentially expressed genes (DEGs) were screened using DESeq (Anders & Huber, 2010). Because there is no reference genome for alfalfa, unigenes with a fold change (FC) ≥ 1 and False Discovery Rate (FDR) < 0.01 were considered as DEGs. The *p* values from RNA-seq were adjusted using the Benjamini–Hochberg method for controlling the FDR (Benjamini & Hochberg, 1995). A volcano plot was constructed to show the relationship between the FDR and FC. An MA plot was constructed to show the gene expression abundance and overall distribution of multiples in two groups of samples. A heat map was constructed for cluster analyses. Homologous classification of DEGs was performed using COG. GO enrichment analyses of DEGs were performed among different groups using topGO software. KEGG enrichment was performed to predict the pathways of DEGs.

### Real-time quantitative PCR validation

Total RNA from each sample was extracted using the method described above and a reverse Transcription Kit (Takara, Tokyo, Japan) was used for first-strand cDNA synthesis. All primers designed using Primer Premier 5.0 (Premier Biosoft International, Palo Alto, CA, USA). The range of primer length is 15∼30 bp. The search range of sense primer and anti-sense primer are 280∼300 bp. The PCR product size is 100∼130 bp. All primers for qPCR are shown in Table S10. The alfalfa *GAPDH* was selected as an internal gene. Each 50 µL reaction contained 0.2 µM of each primer, a 25 µL UltraSYBR Mixture (CWBO, Jiangsu, China) and 5 ng cDNA. The BioRad C1000 server system was used to perform quantitative PCR (qPCR). The PCR reaction procedure consisted of 35 cycles of 95 °C for 15 s, 60 °C for 1 min, and then melting curve analyses. Each reaction was done with four technical duplicates. The relative expression level of each gene was calculated using the 2^−ΔΔCt^ method (Livak & Schmittgen, 2001).

## Results

### Experimental definition

**Figure 1:**
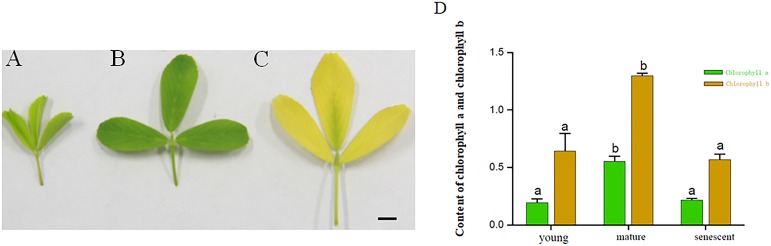
(A–C) represents young, mature and senescent leaves. Bar = 2 cm; (D) Chlorophyll a and Chlorophyll b content in young, mature and senescent leaves. Green represents chlorophyll a. Dark yellow represents chlorophyll a. Lowercase letters a–b represents a significant difference. Error bars represent the SD from three technical replicates.

The results exhibited that the contents of Chlorophyll a and Chlorophyll b in mature leaves were higher than those in the senescent and young leaf stages. The contents of Chlorophyll a in young leaf stages were higher than senescent leaf stages. However, the Chlorophyll a and Chlorophyll b did not change significantly in young and senescent leaf stages. Based on the results above, the young, mature and senescent leaves were selected for RNA-seq (Figs. 1A–1D).

### RNA sequencing and transcriptome assembly

A total of 61.61 G clean data were obtained from RNA-Seq. Each sample dataset included an average of 6.28 G clean data. The Phred-like quality value of the Q30 level showed greater than 93% clean data (Table S1A). A transcript consisting of 500–1000 nucleotides (nt) (transcript number: 42,767) was the largest from the transcript sequences (Fig. S1), followed by 1000–2000, 200–300, and 300–500 nt. The N50 length included 1,502 transcripts. The mean transcript length was 986.36 bp. A transcript with 200–300 nt length of unigene was the highest including 25,779, followed by 500–1000, 300–500, and 1000–2000 nt (Fig. S1). The mean unigene length was 778.88 bp. After transcript assembly, a total of 163,511 transcripts and 77,901 unigenes were obtained.

### Unigene annotation

To classify the potential functions of unigenes, a total of 43,691 unigenes were annotated using one or more of the COG, GO, KEGG, KOG, Pfam, Swissprot, eggNOG, and Nr databases (Table S2). A total of 41,223 unigenes were annotated in Nr (Fig. S2A). The homology between alfalfa and *M. truncatula* was highest, accounting for 67.73%. A total of 12,494 unigenes were analyzed against COG and classified into 25 COG categories (Fig. S2B). Specifically, the category for ‘general function prediction only’ represented the largest group, accounting for 17.79% (3,098), followed by ‘replication, recombination, and repair’ (1,558, 8.94%), ‘transcription’ (1,430, 8.21%), ‘translation, ribosomal structure, and biogenesis’ (1,396, 8.01%) and ‘signal transduction mechanisms’ (1,199, 6.88%). Moreover, ‘energy production and conversion’ (1,015, 5.83%)’ and ‘carbohydrate transport and metabolism’ (1,013, 5.82%) were also found to be involved in alfalfa leaf senescence.

To obtain the functional annotations of unigenes from alfalfa, GO functional classification was performed. A total of 26,741 unigenes were assigned into 55 GO terms at the second level (Table 1, Fig. S3 and Table S3). In the cellular component (CC) group, ‘cell’ (10,219, 6.598%) and ‘cell part’ (10,219, 6.598%) represented the largest clusters, followed by ‘organelle’ (7,584, 4.896%), ‘membrane’ (5,871, 3.790%) and ‘organelle part’ (3,527, 2.277%). In the molecular function (MF) category, ‘catalytic activity’ (14,978, 9.67%) was the most abundant cluster, followed by ‘binding’ (14,930, 9.639%), ‘transporter activity’ (1,695, 1.094%) and ‘structural molecule activity’ (882, 0.569%). In the biological process (BP) category, ‘metabolic process’ (18,697, 12.071%) represented the largest cluster, followed by ‘cellular process’ (15,572, 10.054%), ‘single-organism process’ (12,749, 8.231%), and ‘response to stimulus’ (5,402, 3.488%). In addition, ‘transporter activity,’ ‘enzyme regulator activity,’ ‘biological regulation,’ and ‘guanyl-nucleotide exchange factor activity’ were also found in the alfalfa transcriptome.

**Table 1:** Top 10 gene ontology function classification.

**GO classify 1**	**GO classify 2**	**Unigene**	**Percentage**
Cellular component	Extracellular region	534	0.345%
	Cell	10,219	6.598%
	Membrane	5,871	3.790%
	Cell junction	416	0.269%
	Membrane-enclosed lumen	605	0.391%
	Macromolecular complex	2,987	1.928%
	Organelle	7,584	4.896%
	Organelle part	3,527	2.277%
	Membrane part	3,197	2.064%
	Cell part	10,219	6.598%
Molecular function	Nucleic acid binding transcription factor activity	679	0.438%
	Catalytic activity	14,978	9.670%
	Receptor activity	120	0.077%
	Structural molecule activity	882	0.569%
	Transporter activity	1,695	1.094%
	Binding	14,930	9.639%
	Electron carrier activity	642	0.414%
	Antioxidant activity	220	0.142%
	Enzyme regulator activity	289	0.187%
	Molecular transducer activity	306	0.198%
Biological process	Metabolic process	18,697	12.071%
	Cellular process	15,572	10.054%
	Signaling	1,681	1.085%
	Multicellular organismal process	2,193	1.416%
	Developmental process	2,227	1.438%
	Single-organism process	12,749	8.231%
	Response to stimulus	5,402	3.488%
	Localization	3,795	2.450%
	Biological regulation	5,177	3.342%
	Cellular component organization or biogenesis	3,047	1.967%

KEGG classification was performed to identify the unigene functions in terms of biological system networks. A total of 14,550 unigenes were successfully assigned to 129 KEGG pathways (Fig. S2C). All were divided into five categories: cellular processes, environmental information processing, genetic information processing, metabolism and organism systems. ‘Ribosome’ [ko03010] (734 unigenes, 4.8%) was the most abundant pathway, followed by ‘carbon metabolism’ [ko01200] (625 unigenes, 4.09%), ‘biosynthesis of amino acids’ [ko01230] (517 unigenes, 3.38%), and ‘protein processing in endoplasmic reticulum’ (454, 2.97%).

### DEG identification and functional annotation in young, mature, and senescent leaves

A total of 5,133 (—log_2_FC—>1) were identified from the RNA-Seq of young, mature, and senescent alfalfa leaves. A comparison between mature and young leaves showed that 451 DEGs were involved in these two biological processes. Young leaves were a reference group.

**Figure 2:**
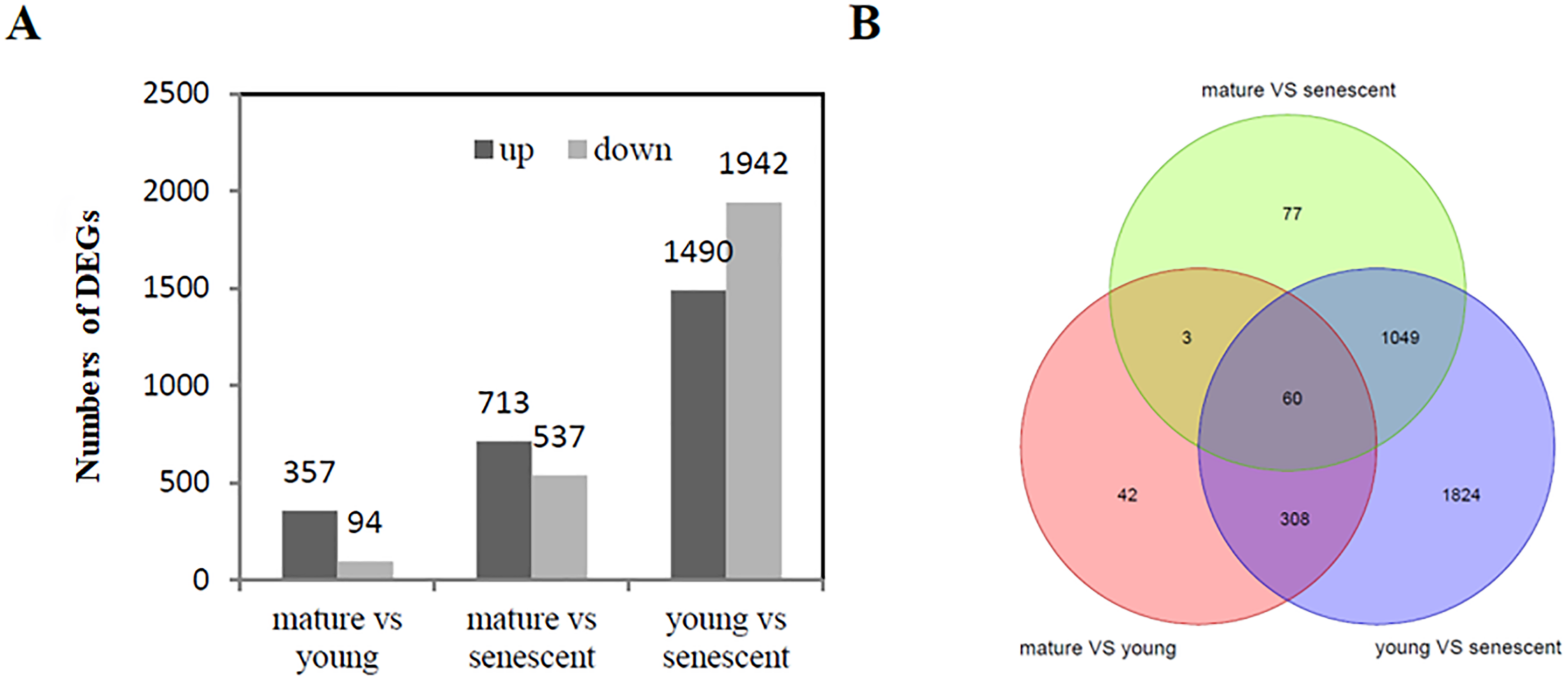
DEG analysis (A) The histogram shows the up and down regulated DEGs among young, mature and senescent leaves; (B) the Venn diagram shows the distribution of DEGs among young, mature and senescent leaves. The light green represents the mature vs. senescent group; the light purple represents the young group.

Specifically, 357 genes were up-regulated, and 94 genes were down-regulated (Fig. 2A, Fig. S4A). A comparison between mature and senescent leaves showed that 1,250 DEGs were involved in the maturation and senescence development processes, including 713 up-regulated genes and 537 down-regulated genes. Mature leaves were a reference group (Fig. 2A, Fig. S4B). Moreover, a total of 3,432 DEGs were identified in young and senescent leaves, which represented the most abundant DEG groups, and included 1,490 up-regulated and 1,942 down-regulated DEGs. Young leaves were a reference group (Fig. 2A, Fig. S4C). The Venn diagram showed that 60 DEGs were found overall (Fig. 2B). All DEGs were subjected to GO enrichment analyses, which revealed 7,529 GO terms. A comparison between young and senescent leaf groups (Table S4) showed that the GO terms ‘protein phosphorylation’ (GO: 0006468) and ‘isopentenyl diphosphate biosynthetic process, methylerythritol 4-phosphate pathway’ (GO: 0019288) were the most enriched in the BP category (Fig. S5A). The GO terms ‘chloroplast thylakoid membrane’ (GO: 0009535), ‘chloroplast stroma’ (GO: 0009570,) and ‘chloroplast envelope’ (GO: 0009941) were the most enriched in the CC category (Fig. S5B). The GO term ‘protein serine/threonine kinase activity’ (GO: 0004674) was the most enriched in the MF category. A comparison between the mature and young leaf groups (Table S5) showed that the GO term ‘protein phosphorylation’ (GO: 0006468) was the most enriched in the BP category (Fig. S6A). The GO terms ‘Cul4-RING E3 ubiquitin ligase complex’ (GO: 0080008), ‘ubiquitin ligase complex’ (GO: 0000151) and ‘integral component of membrane’ (GO: 0016021) were the most enriched in the CC category (Fig. S6B). The GO term ‘protein serine/threonine kinase activity’ (GO: 0004674) was the most enriched in the MF category (Fig. S6C). GO enrichment analyses between the mature and senescent groups (Table S6) showed that the GO term ‘protein phosphorylation’ (GO: 0006468) was the most enriched in the BP category (Fig. S7A). The GO term ‘chloroplast thylakoid membrane’ (GO: 0009535) was the most enriched in the CC category (Fig. S7B). The GO term ‘protein serine/threonine kinase activity’ (GO: 0004674) was the most enriched in the MF category (Fig. S7C).

### Carbon metabolism, starch and sucrose metabolism and biosynthesis of amino acids pathways

Several metabolic pathways were identified by KEGG enrichment analysis in young and mature leaves. A total of 14 DEGs were enriched in the carbon metabolism (ko01200) pathway (Table 2), which is the most significantly metabolic pathway. Interestingly, all DEGs were down-regulated in this pathway. Among the 14 DEGs, the enzyme gene was dominant, including *Glyceraldehyde-3-phosphate dehydrogenase* (*GAPCP1*), *Phosphoserine aminotransferase* (*PSAT1*), *Malate dehydrogenase* (*MDH*) and *NADP-dependent malic enzyme* (*ME1*). In addition, eight DEGs were enriched in the starch and sucrose metabolism (ko00500) pathway. Three DEGs were up-regulated and five DEGs were down-regulated. Among these unigenes, *Beta-amylase 3* and *Probable fructokinase-7* had high expression levels. Finally, a total of 11 DEGs (one up-regulated DEG and ten down-regulated DEGs) were enriched in the biosynthesis of amino acids (ko01230). *Gamma-glutamyl phosphate reductase* was highly expressed.

**Table 2:** Significantly enriched pathways in alfalfa during young and mature stages.

**KEGG pathway name**	**KEGG id**	***P*-value**	**Corrected *P*-value**	**DEG up- regulated number**	**DEG down- regulated number**
Carbon metabolism	ko01200	8.60E-03	5.42E-01	0	14
Starch and sucrose metabolism	ko00500	2.53E-04	1.59E-02	3	5
Biosynthesis of amino acids	ko01230	2.73E-02	1	1	10

### Ribosome, photosynthesis and phenylpropanoid biosynthesis pathways

We also found some metabolic pathways in mature and senescent leaves (Table 3). First, a total of 32 DEGs (14 up-regulated DEGs and 18 down-regulated DEGs) were enriched in the ribosome pathway (ko03010). In this pathway, the ribosomal protein is dominant, and includes 30S (*RPS13*, *RPS9*, *RPS17*), 40S (*RPS14*, *RPS26C*, *RPS9B*), 50S (*RPL18*, *RPL29*, *RPL9*) and 60S (*T25B24.7*, *RPL39*, *RPP2B*). Second, 30 DEGs were enriched in the photosynthesis (ko00195) pathway. Interestingly, all DEGs were down-regulated in the photosynthesis pathway, of which 18 genes, including *PSAN*, *PSAF* and *PSBY,* were associated with photoreaction,. Three ATP synthase and three Oxygen-evolving enhancer protein genes were also identified. Finally, 24 DEGs (22 up-regulated and two down-regulated) were enriched in the phenylpropanoid biosynthesis pathway (ko00940). Two *Cyanogenic beta-glucosidase*, three *Isoliquiritigenin 2-O-methyltransferase* and one *Peroxidase* were expressed at high levels in the phenylpropanoid biosynthesis pathway.

**Table 3:** Significantly enriched pathways in alfalfa during mature and senescent stages.

**KEGG pathway name**	**KEGG id**	***P*-value**	**Corrected *P*-value**	**DEG up- regulated number**	**DEG down- regulated number**
Ribosome	ko03010	1.16E-01	1	14	18
Photosynthesis	ko00195	3.23E-13	3.00E-11	0	30
Phenylpropanoid biosynthesis	ko00940	8.39E-06	7.80E-04	22	2

### Comparison of senescence associated genes between alfalfa and other plants

**Figure 3:**
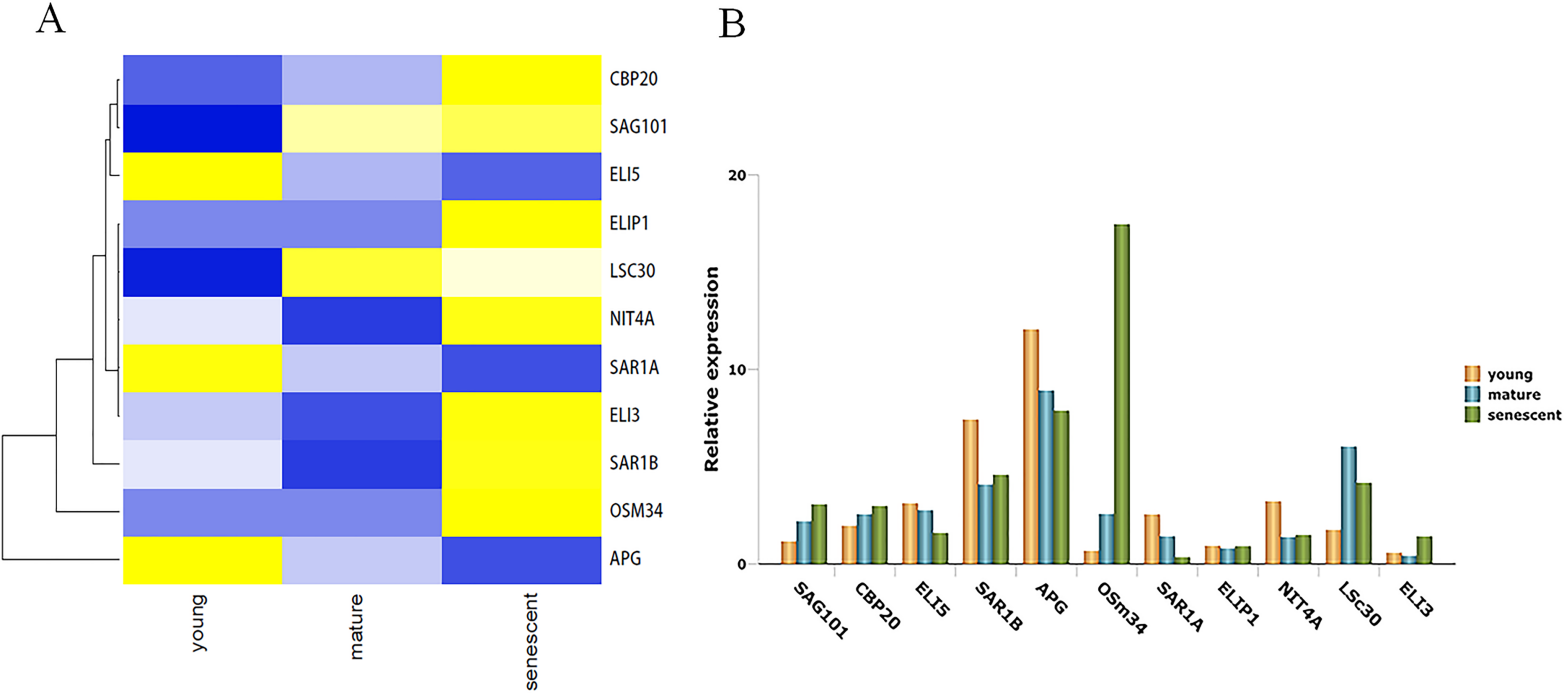
The unigenes linked to leaf senescence in *M. sativa*. (A) Heat map shows 11 DEGs clustering at different transcriptome databases. (*X*-axis) represents different databases including young, mature and senescent. (*Y*-axis) represents unigene name. The different colors represent FPKM of DEGs. Blue to yellow represents −1 to 1. (B) Histogram shows the 11 DEGs relative expression levels of young, mature and senescent databases.

Leaf senescence is a complex process that involves gene regulation. In order to identify senescence genes between alfalfa and other plant species, all DEGs were aligned using BLASTN searches in the National Center for Biotechnology Information (NCBI). Interestingly, 11 were identified as senescence genes. Most senescence genes were homologous to genes in *Arabidopsis thaliana*, followed by *Brassica napus* and *Nicotiana tabacum*. Among these genes, *SAG101*, *nuclear cap-binding protein subunit 2* (*CBP20*) and *osmotin-like protein 34* (*OSM34*) were up-regulated in all of the transcriptome databases. Moreover, *probable mannitol dehydrogenase5* (*ELI5*), GTP-binding protein (*SAR1A*) and *GDSL esterase/lipase* (*APG*), were down-regulated in all groups. It is important to point out that *APG* was down-regulated more than four-fold in senescent leaves compared with young leaves. *OSM34* was up-regulated more than five-fold in mature and senescent groups (Table S7). All genes related to leaf senescence were clustered together, as shown in Fig. 3A. To further verify the trends in relative gene expression, 11 genes were subjected to qPCR validation. As shown in Fig. 3B, a similar clustered trend was observed. GO classification showed that *ELI3* was involved in the oxidation–reduction process (GO: 0055114). KEGG enrichment showed that *NIT4A* was involved in the beta-cyano-L-alanine hydratase/nitrilase pathway.

### Cluster of DEGs enriched in several biological processes

**Figure 4:**
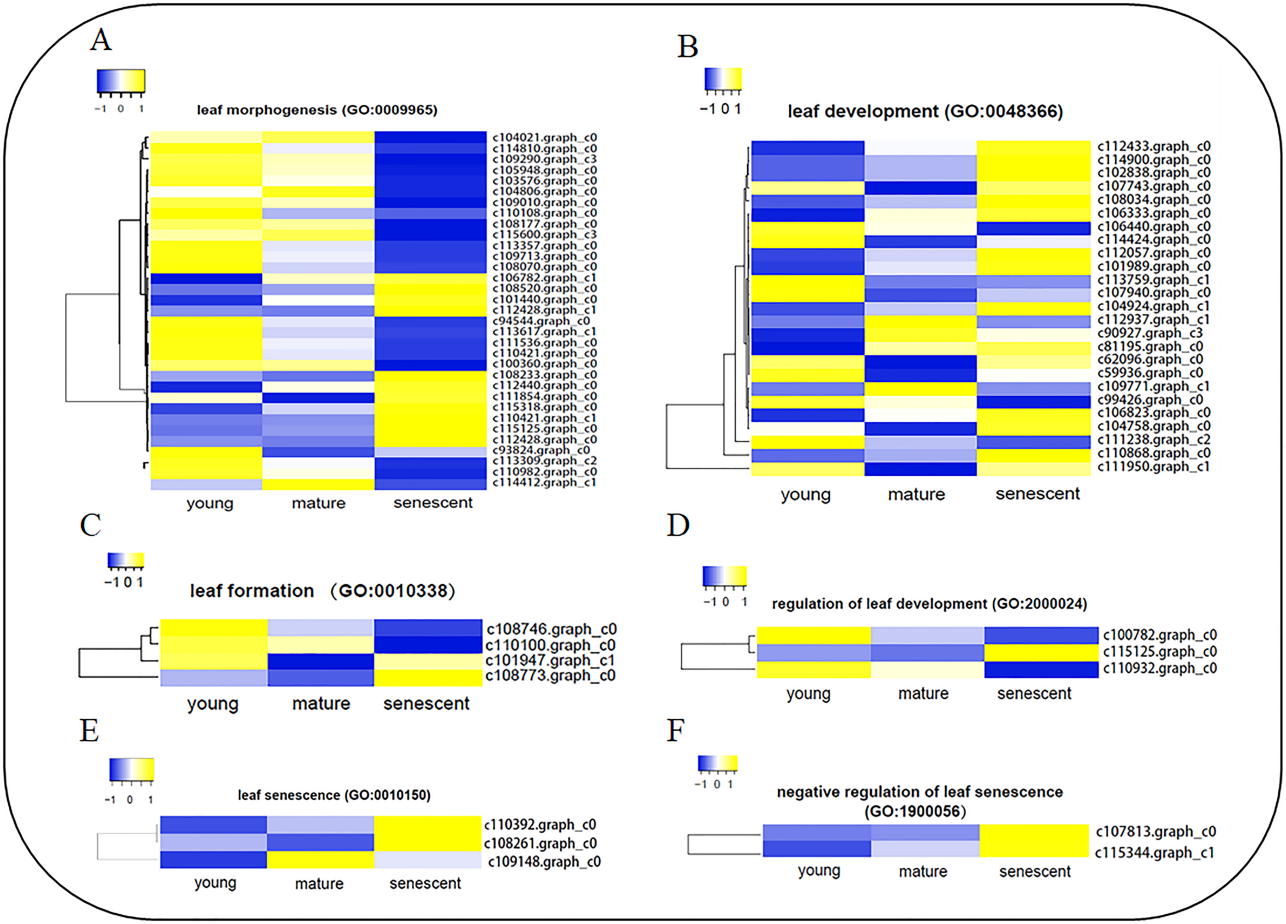
The heat map showing FPKM of DEGs involved in six biological processes by GO enrichment analysis. (A) leaf morphogenesis (GO:0009965); (B) leaf development (GO:0048366); (C) leaf formation (GO:0010338); (D) regulation of leaf development (GO:2000024); (E) leaf senescence (GO:0010150); (F) negative regulation of leaf senescence (GO: 1900056). The clustering method is average linkage. The distance measurement method is Euclidean. Blue to yellow represents −1 to 1.

To identify DEGs involved in leaf senescence development, all DEGs were subjected to GO enrichment analyses. Interestingly, six clusters of DEGs were identified during stages of leaf senescence development (Fig. 4, Table S8a), including leaf morphogenesis (GO:0009965), leaf development (GO:0048366), leaf formation (GO:0010338), regulation of leaf development (GO:2000024), leaf senescence (GO:0010150) and negative regulation of leaf senescence (GO:1900056). A total of 34 DEGs were enriched in the leaf morphogenesis process (Fig. 4A). Several enzyme genes play active roles, such as *Peptidyl-prolyl cis-trans isomerase* (*CYP71*), *Receptor-like serine/threonine-protein kinase* (*ALE2*) and *Homogentisate solanesyltransferase* (*MEC18*). Additionally, seven DEGs were novel genes in the leaf morphogenesis process. A total of 25 DEGs were enriched in the leaf development process (Fig. 4B). Several new genes were annotated, such as *Pyrophosphate-energized vacuolar membrane proton pump* (*c111950.graph_c1*), *Chromatin assembly factor 1 subunit* (*MUB3.9*) and *Tubulin gamma-1 chain* (*TUBG1*). In addition, two DEGs were novel genes in the leaf development process. In the leaf senescence process (Fig. 4E), three DEGs were enriched, including *Aminotransferase* (*ALD1*), *Phospholipid–sterol O-acyltransferase* (*F21M11.5*) and *c109148.graph_c0* (no annotation). Interestingly, *Transcription factor JUNGBRUNNEN 1* (*F23E6.1*) and *U11/U12 small nuclear ribonucleoprotein* (*SNRNP59*) were enriched in the negative regulation of the leaf senescence process (Fig. 4D). In the leaf formation process (Fig. 4C), four DEGs were enriched. Interestingly, *Probable auxin efflux carrier component 1* (*c101947.graph_c1*) and *Auxin efflux carrier component 1* (*c110100.graph_c0*) were shown to regulate leaf formation. Three elongator complex protein encoding genes were identified in the regulation of the leaf development process (Fig. 4F).

### TFs involved in leaf senescence

**Figure 5:**
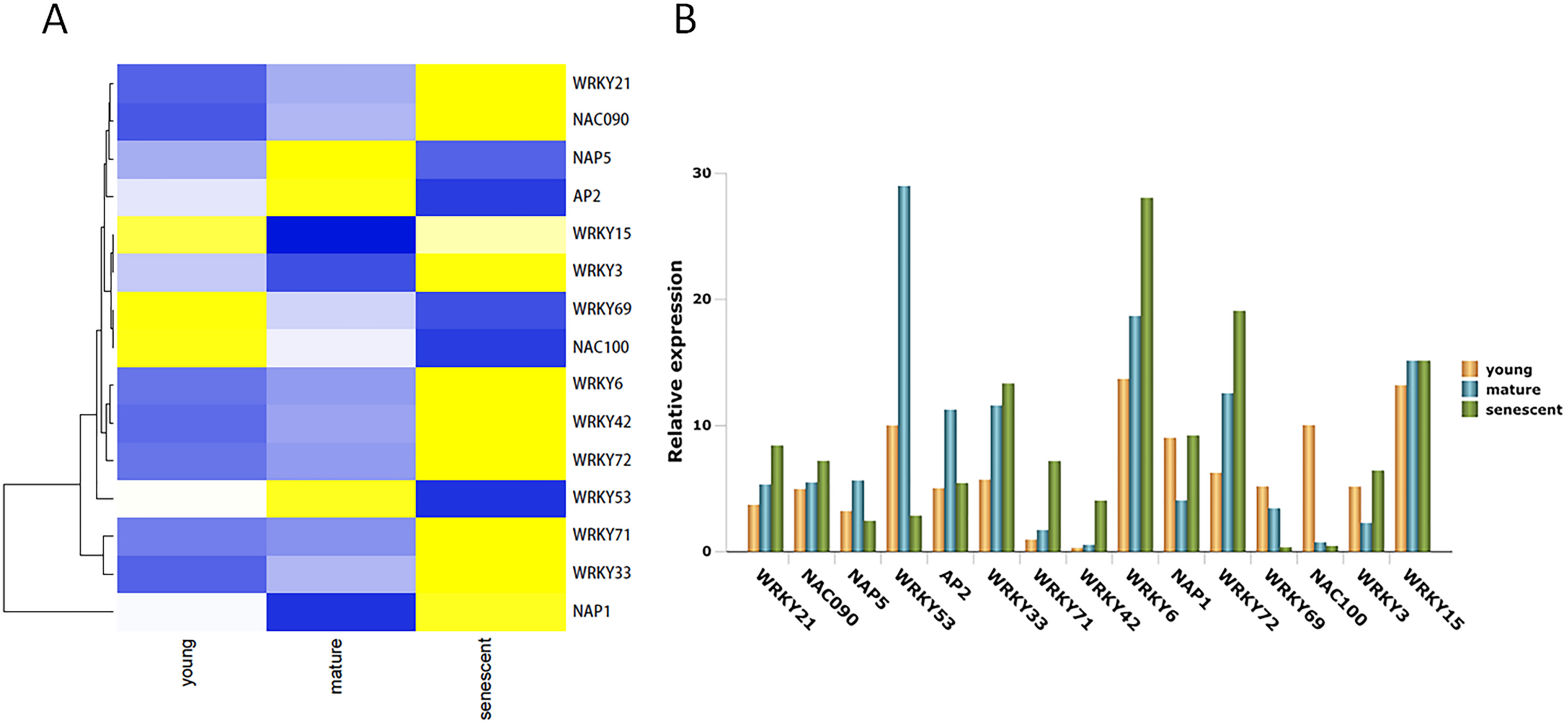
The TFs associated with leaf senescence in *M.sativa*. (A) Heat map shows 15 DEGs clustering at different transcriptome databases. (*X*-axis) represents different databases including young, mature and senescent. (*Y*-axis) represents unigene name. Blue to yellow represents −1 to 1. (B) Histogram shows the 15 DEGs relative expression levels of young, mature and senescent databases.

TFs play many biological regulatory roles in plants, as well as an irreplaceable role in leaf senescence. In this study, 15 DEGs were identified from the alfalfa transcriptome (Table S9). Seven DEGs were up-regulated in young, mature, and senescent leaves, and included *NAC domain-containing protein 090* (*NAC090*), *probable WRKY transcription factor 21* (*WRKY21*), *WRKY42*, *WRKY72*, *WRKY33*, *WRKY71*, and *WRKY6* (Fig. 5A). *NAC100* and *WRKY69* were down-regulated in all leaves. *Nucleosome assembly protein 1* (*NAP1*), *WRKY3,* and *WRKY15* were down-regulated in young and mature leaves, and up-regulated in mature and senescent leaves. *NAP5*, *WRKY53,* and *AP2* were up-regulated in young and mature leaves, and down-regulated in mature and senescent leaves. All TFs were validated using qPCR (Fig. 5B). The relative expression trends of the TFs were consistent with gene clustering. GO classification showed that *WRKY15* was involved in the ‘respiratory burst’ involved in ‘defense response’ (GO: 0002679) in the MF category.

### The qPCR validation

**Figure 6:**
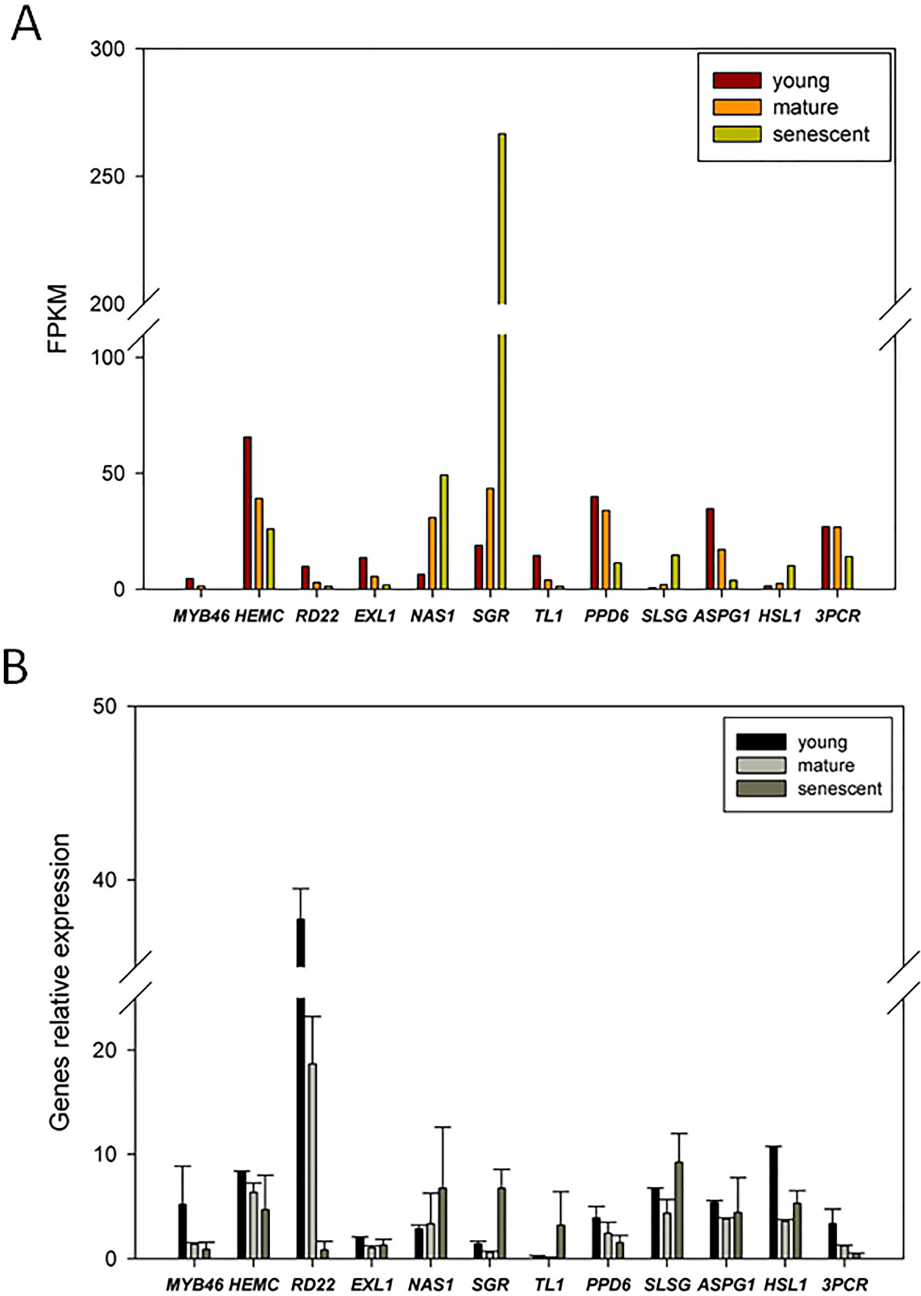
qRT-PCR validation of DEGs from the young, mature and senescent RNA-seq databases in alfalfa; (A) the DEGs transcript abundance changes calculated by FPKM method; (B) DEGs relative expression levels of young, mature and senescent leaves validated by qPCR. All DEGs were performed with three technical repetitions; the calculated methods use 2^−ΔΔCt^.

To confirm the expression patterns of DEGs from RNA-Seq, we randomly selected 12 candidate DEGs for qPCR analyses. The transcript abundance changes in DEGs from young, mature, and senescent leaves are shown in Fig. 6A. The qPCR results indicated that *nicotianamine synthase 1* (*NAS1*) and *stay-green* (*SGR*) were up-regulated in young and mature leaves (Fig. 6B). Six DEGs were up-regulated in mature and senescent leaves, and included *NAS1*, *SGR*, *thaumatin-like protein 1* (*TL1*), *S-locus-specific glycoprotein* (*SLSG*), *aspartic protease in guard cell 1* (*ASPG1*) and *receptor-like protein kinase* (*HSL1*). In young and mature leaves, ten DEGs were down-regulated, including *MYB46*, *porphobilinogen deaminase*, *chloroplastic* (*HEMC*), *dehydration-responsive protein* (*RD22*), *esterase/lipase* (*ELX1*), *TL1*, *PsbP domain-containing protein 6* (*PPD6*), *HSL1*, *ASPG1*, *HSL1*, and *protochlorophyllide reductase* (*3PCR*). *MYB46*, *HEMC*, *RD22*, *ELX1*, *PPD6,* and *3PCR* were down-regulated in mature and senescent leaves. In general, the relative expression levels of the ten DEGs were consistent with the RNA-Seq results. However, *TL1* and *ASPG1* were up-regulated in mature and senescent leaves, and the transcript abundance of *TL1* was down-regulated. The differences between qPCR and RNA-Seq might be caused by unexpected RNA-Seq errors. However, most expression patterns of DEGs identified from RNA-Seq can be confirmed by qPCR, showing that RNA-Seq is an efficient, accurate and high-throughput tool for DEGs analyses of leaf senescence in alfalfa.

## Discussion

Leaf senescence is a developmentally programmed degeneration process controlled by genetic factors *in vivo* and by environmental signals *in vitro* (Lim, Woo & Nam, 2003). Previous studies have shown that 3,624 DEGs are involved in leaf development in cotton, including 50 TF families (Lin et al., 2015). RNA-seq analyses were used to investigate flag leaf development, senescence, and mineral dynamics in switchgrass, and the results revealed that NAC TFs were significantly upregulated in senescing flag leaves (Palmer et al., 2015). However, the molecular mechanisms in young, mature, and senescent flag leaves in alfalfa are unclear. Therefore, RNA-seq was applied and 5,133 DEGs from these leaves were identified. Several DEGs were directly involved in leaf development and senescence. For example, six clusters of DEGs were identified that were involved in leaf morphogenesis, leaf development, leaf formation, regulation of leaf development, leaf senescence and negative regulation of leaf senescence biological process by GO enrichment. Moreover, ribosome and phenylpropanoid biosynthesis pathways and starch and sucrose metabolism pathways were identified as being involved in leaf development and senescence in alfalfa by KEGG enrichment. Transcriptome analysis suggested that several novel genes were obtained. such as *c111950.graph_c1*, *MUB3.9* and *TUBG1*.

### Several metabolic pathways regulate leaf development and senescence

Several specific transcriptional signatures of cell type can predict previously unknown cellular functions. Dominant expression patterns were identified that found a computational pipeline. However, several disparate cell types have similarly transcriptional traits (Brady et al., 2007). In this study, a cluster of 32 DEGs were involved in the ribosome pathway (ko03010) in mature and senescent leaf groups. Most members regulate ribosomal protein genes. Previous studies have shown that ribosomal proteins are encoded by two to seven gene family members. *Arabidopsis thaliana* has 80 cytoplasmic ribosomal proteins (Barakat et al., 2001; Byrne, 2009). The ribosomal protein genes have differential expression in different tissue. For example, one copy of *RPS5* ribosomal protein genes is expressed at high levels in *Arabidopsis thaliana* shoot and root meristem, whereas the other copy is expressed at lower levels in the shoot and root meristem (Weijers et al., 2001). Our study suggested that *RPS13*, *RPS9* and *RPS17* were identified in mature and senescent leaves. These ribosomal protein genes are expressed at high levels in mature leaves and are expressed at lower levels in young and senescent leaves. These results indicated that novel ribosomal protein genes can have a similar transcriptional trait in specific cell types in alfalfa.

Carbon metabolites control photosynthesis feedback regulation of leaf development and senescence (Paul & Pellny, 2003). The excessive accumulation of Pi inhibits metabolic flow (Dumschott et al., 2017). This article shows that 14 DEGs are involved in the carbon metabolism pathway in young and mature leaves (Table 2). These DEGs, such as *GAPCP1*, *PSAT1* and *ME1,* were down-regulated. These enzyme genes can play feedback regulation roles during leaf development and senescence. Sucrose degradation and starch synthesis also regulate leaf development and senescence via regulatory signals responding to sucrose and oxygen availability (Geigenberger, 2003). Eight DEGs were identified that are involved in starch and sucrose metabolism in young and mature leaves. *Beta-amylase 3* and *Probable fructokinase-7* are expressed at high levels in mature leaves. These novel DEGs can play regulatory roles in the starch and sucrose metabolism pathway.

Phenylpropanoid metabolism via intermediates of the shikimate pathway generates large amounts of secondary metabolites in plants. The shikimate pathway is key to the biosynthesis of phenylpropanoids (Vogt, 2010). The 3-deoxy-D-arabinose-heptulosonate synthase (DHAP) regulated biosynthesis of phenylpropanoids at the transcription level. However, phenylalanine inhibits arogenate and prephenate dehydratase activity (Lynch et al., 2017; Yamada et al., 2008). In our study, two cyanogenic beta-glucosidase, three isoliquiritigenin 2-O-methyltransferase and one peroxidase are involved in the phenylpropanoid biosynthesis pathway. These enzyme genes were highly expressed in the mature and senescent leaf group. In summary, the phenylpropanoid biosynthesis pathway might play roles during leaf development and senescence.

### Comparison of SAG between alfalfa and other plants

SAGs are preferentially expressed during leaf senescence. SAGs encode proteins involved in many signal transduction pathways including macromolecule degradation, induction of defense mechanisms, and signaling and regulatory events (Gepstein et al., 2003). SAGs have been identified in a wide variety of species including *Brassica napus*, *Arabidopsis*, and rice (Buchanan-Wollaston, 1994; Nam, 1997; Lee et al., 2001). In this study, 11 SAGs were identified during leaf development and senescence in alfalfa. It has been previously reported that over 70 SAGs participate in the senescence process in *Arabidopsis* (Gepstein et al., 2003). Fourteen SAGs are involved in dark-induced and natural leaf senescence in rice (Lee et al., 2001). *SAG113* is a Golgi-localized PP2C family protein phosphatase that is induced by ABA in senescing *Arabidopsis* leaves, and is a negative regulator of ABA signal transduction (Zhang et al., 2012). *SAG27* is an *NIT2* gene, which encodes the enzyme nitrilase 2. *NIT2* is induced during leaf senescence (Bartel & Fink, 1994). *CBP20* is involved in hormonal and developmental regulation and accumulates in lower source leaves in tobacco (Hensel, Kunze & Kunze, 1999). *AtOSM34* is an osmotin-like protein that is involved in leaf senescence in *Arabidopsis* (Quirino, Normanly & Amasino, 1999). *LSC94* and *LSC222* are expressed during early leaf senescence in *B. napus*. Moreover, *LSC94* and *LSC222* are induced by salicylic acid treatment (Hanfrey, Fife & Buchanan-Wollaston, 1996). This study shows that *SAG101*, *CBP20*, and *OSM34* also identified in whole young, mature, and senescent alfalfa leaves (Fig. 3A), whereas seven novel SAGs are involved in leaf development and senescence in alfalfa (Table S7). These novel SAGs might play regulatory roles during leaf senescence in alfalfa.

### Cluster of DEGs associate with several biological processes in alfalfa

The progression of component cells via proliferation and expansion eventually formed a mature organ. 131 genes were identified in the growing organs of *Arabidopsis* (Beemster et al., 2005). Nine hundred and sixty genes were involved in eight hormone signal transduction pathways during leaf senescence of *Gossypium hirsutum L.* by RNA-Seq (Lin et al., 2015). In this study, six clusters of DEGs were identified that enriched six biological processes during leaf development and senescence. 33 DEGs were enriched in leaf morphogenesis processes (Fig. 4A). Mostly composed of enzyme genes, seven DEGs were novel genes. These genes may regulate early leaf growth in alfalfa. It has been reported previously that the control of cell proliferation is associated with the control of cell size (Tsukaya, 2003). *AGO1* regulated multicellular organisms in *Arabidopsis*. We found 25 DEGs involved in leaf development process (Fig. 4B). *c111950.graph_c1*, *MUB3.9* and *TUBG1* were identified as novel genes in alfalfa. These novel genes might play a role in the mature leaf development process. Interestingly, three DEGs were involved in the leaf senescence process (Fig. 4E), including *ALD1*, *F21M11.5* and *c109148.graph_c0*. However, *F23E6.1* and *SNRNP59* were identified as being involved in the negative regulation of the leaf senescence process (Fig. 4D). *ALD1* plays active defense in *Arabidopsis* (Song et al., 2004; Cecchini et al., 2015). We believe that these genes might play the role of maintaining balance during leaf senescence. Finally, four DEGs were also identified as being involved in leaf formation (Fig. 4C), and three encoded elongator complex protein genes were involved in regulation of leaf development (Fig. 4F). These genes might play a role in specific development in alfalfa.

### TF roles in leaf senescence

WRKY TFs represent a family of plant-specific zinc-finger-type TFs that are involved in senescence and trichome development (Miao et al., 2004). *WRKY53* is involved in a complex TF signaling network that regulates gene expression in senescence (Miao et al., 2004). *AtWRKY6* is influenced by signaling factors involved in the plant defense response and senescence processes (Robatzek & Somssich, 2001). In a previous study, *AtWRKY22* overexpression in a dark treatment accelerated senescence; conversely, *Arabidopsis* knockout lines delayed senescence (Zhou, Jiang & Yu, 2011). *AtWRKY70* is a negative regulator of leaf senescence in *Arabidopsis*. It is involved in defense signaling pathways through salicylic acid-mediated signaling cascades (Ülker, Mukhtar & Somssich, 2007). *WRKY53*, *WRKY54*, and *WRKY70* are involved in the regulatory network that integrates internal and environmental factors to regulate specific expression of leaf senescence genes in *Arabidopsis* (Besseau, Li & Palva, 2012). *OsWRKY23* overexpression in *Arabidopsis* accelerates leaf senescence in a darkness treatment (Jing et al., 2009). The NAC family of genes plays an important role in senescence (Guo, Cai & Gan, 2004; Guo & Gan, 2006). Overexpression of *AtNAP* accelerates precocious senescence in *Arabidopsis* (Guo & Gan, 2006). *NTL4* is a molecular switch that is involved in the metabolism of reactive oxygen species (ROS) to induce leaf senescence in *Arabidopsis* (Lee et al., 2012). In this study, *WRKY21*, *WRKY42*, *WRKY72*, *WRKY33*, *WRKY71*, and *WRKY6* were regulated in young, mature, and senescent leaves. These TFs might accelerate leaf senescence. However, *WRKY53* was down-regulated in such leaves, so it might act as a negative regulator in leaf senescence. Several NAC TFs are also involved in leaf senescence. *NAC090* was up-regulated in all leaf groups and might induce leaf senescence. However, *NAC100* was down-regulated in all stages. *NAC100* might act as a negative regulator in leaf senescence. In addition, *NAP5* and *AP2* TFs are also involved in leaf senescence.

## Conclusions

The molecular characteristics of DEGs in young, mature, and senescent alfalfa leaves were identified using transcriptome analyses. A total of 163,511 transcripts and 77,901 unigenes were identified from the transcriptomes. Among all of the unigenes, 5,133 were differentially expressed. KEGG enrichment analyses revealed that ribosome, phenylpropanoid biosynthesis pathways and starch and sucrose metabolism pathways are involved in leaf development and senescence in alfalfa. *RPS13*, *RPS9* and *RPS17* can play a regulatory role in ribosome pathway. GO enrichment analyses exhibited that six clusters of DEGs were identified as being involved in leaf morphogenesis, leaf development, leaf formation, regulation of leaf development, leaf senescence and negative regulation of leaf senescence biological process. Seven novel genes were identified in the leaf morphogenesis process. Moreover, TFs, mainly those in the WRKY and NAC families, also participated in leaf senescence. Our results provide valuable information for interpreting the molecular mechanisms of leaf development and senescence in alfalfa.

##  Supplemental Information

10.7717/peerj.8426/supp-1 Supplemental Information 1 Supplemental Figures and Tables

## Additional Information and Declarations

### Data Availability

The following information was supplied regarding data availability: The data is available at GenBank: SRP149659.
