# Supplementary material for: Global transcriptome analysis of alfalfa reveals six key biological processes of senescent leaves
Source: PeerJ. 2020 Jan 21;8:e8426. doi: 10.7717/peerj.8426 (PMC6979412; doi:10.7717/peerj.8426)
Supplement: Supplemental Information 1 [file peerj-08-8426-s001.zip › peerj-37563-supplemental_data/supplemental data/figure S1-S7/Figure. S2.pptx]

## Slide 1
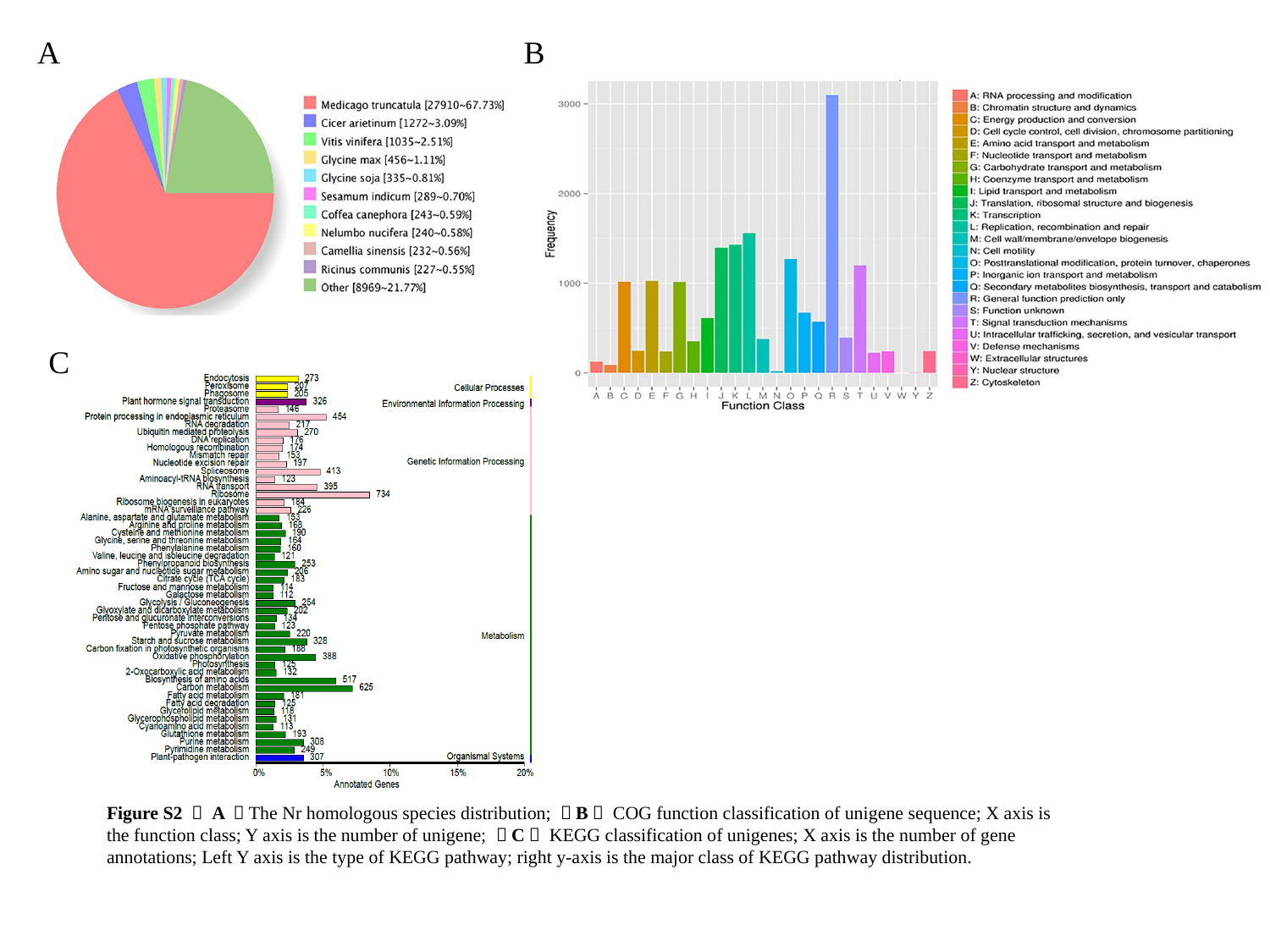

A
B
C
Figure S2 （ A ）The Nr homologous species distribution; （B） COG function classification of unigene sequence; X axis is the function class; Y axis is the number of unigene; （C） KEGG classification of unigenes; X axis is the number of gene annotations; Left Y axis is the type of KEGG pathway; right y-axis is the major class of KEGG pathway distribution.
