# Supplementary material for: Global transcriptome analysis of alfalfa reveals six key biological processes of senescent leaves
Source: PeerJ. 2020 Jan 21;8:e8426. doi: 10.7717/peerj.8426 (PMC6979412; doi:10.7717/peerj.8426)
Supplement: Supplemental Information 1 [file peerj-08-8426-s001.zip › peerj-37563-supplemental_data/supplemental data/Table S1-S11/Table S1a.docx]

| Term | Transcript | Unigene |
| --- | --- | --- |
| N50 Length | 1,502 | 1,298 |
| Mean Length | 986.36 | 778.88 |
| Total Length | 160,294,740 | 60,675,242 |
| Total Number | 162,511 | 77,901 |

**Table S1a** RNA sequenccing assembly result statistics
