# Supplementary figures and images for: Global transcriptome analysis of alfalfa reveals six key biological processes of senescent leaves
Source: PeerJ. 2020 Jan 21;8:e8426. doi: 10.7717/peerj.8426 (PMC6979412; doi:10.7717/peerj.8426)

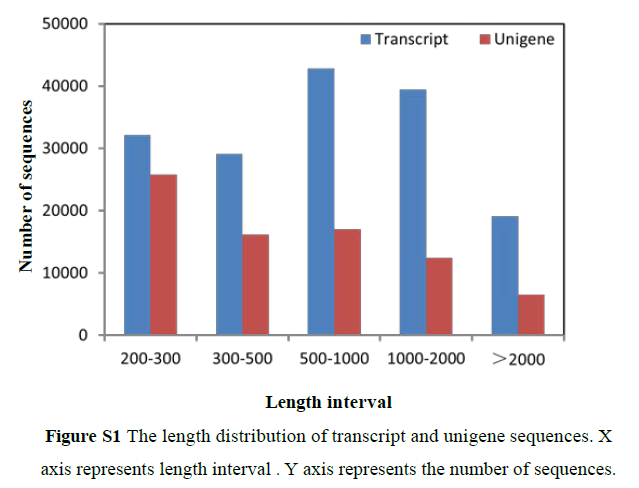

Supplement: Supplemental Information 1 [file peerj-08-8426-s001.zip › peerj-37563-supplemental_data/supplemental data/figure S1-S7/Figure S1.png]

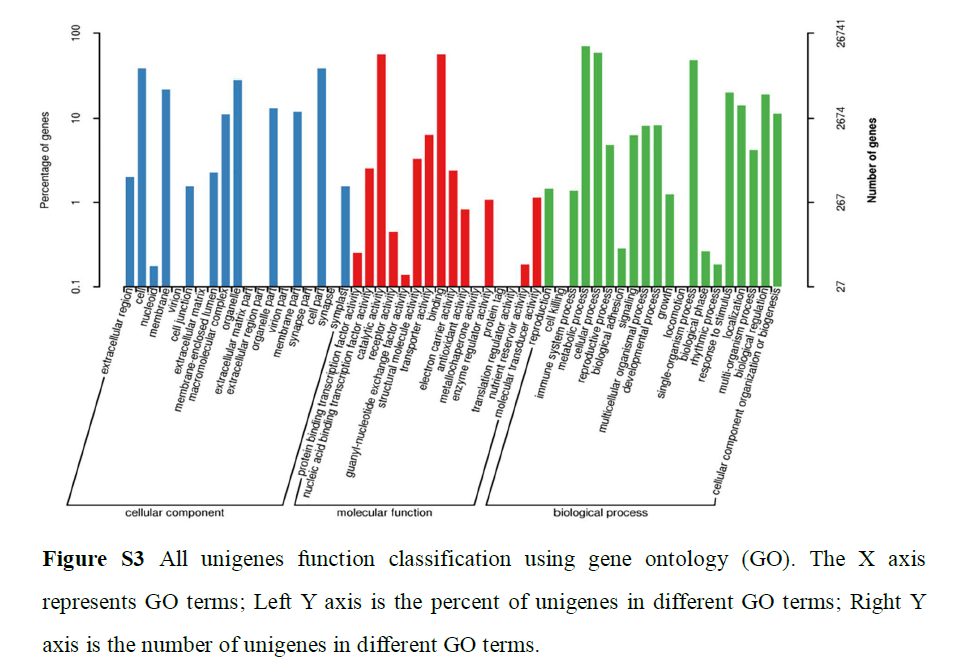

Supplement: Supplemental Information 1 [file peerj-08-8426-s001.zip › peerj-37563-supplemental_data/supplemental data/figure S1-S7/Figure S3.png]

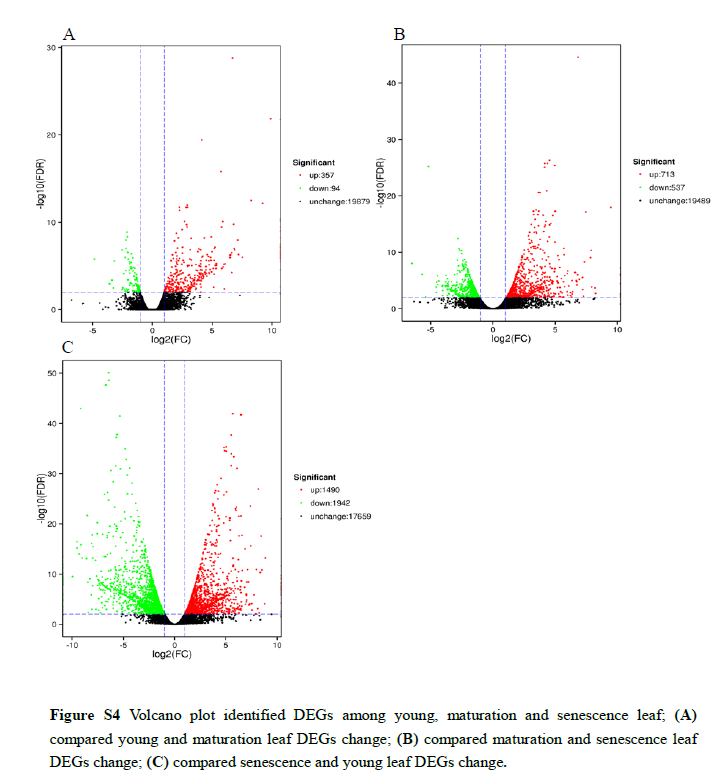

Supplement: Supplemental Information 1 [file peerj-08-8426-s001.zip › peerj-37563-supplemental_data/supplemental data/figure S1-S7/Figure S4.png]

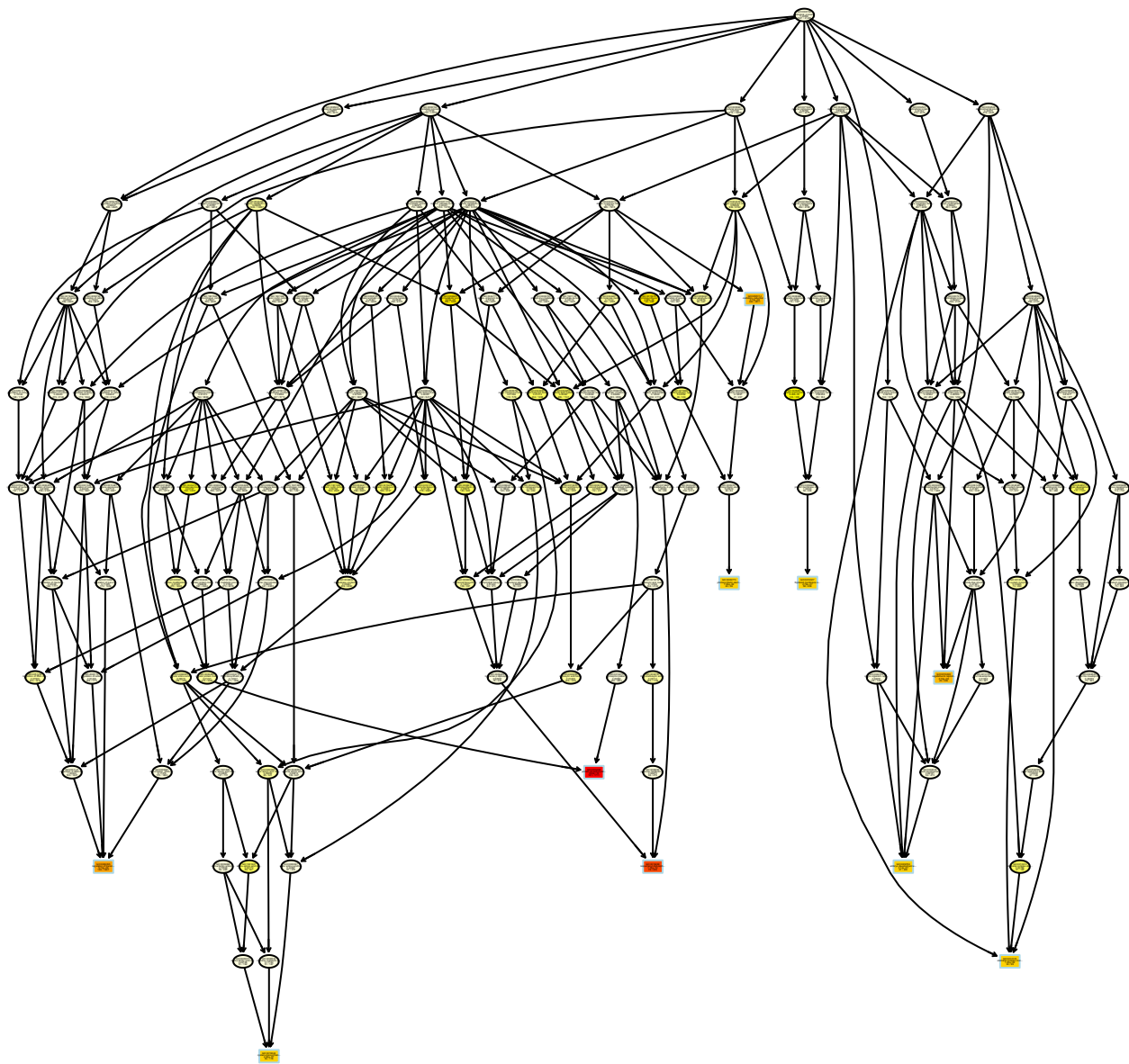

**Figure S5A.** GO enrichment analysis in BP of young and senescent groups

Supplement: Supplemental Information 1 [file peerj-08-8426-s001.zip › peerj-37563-supplemental_data/supplemental data/figure S1-S7/Figure S5A .pdf]

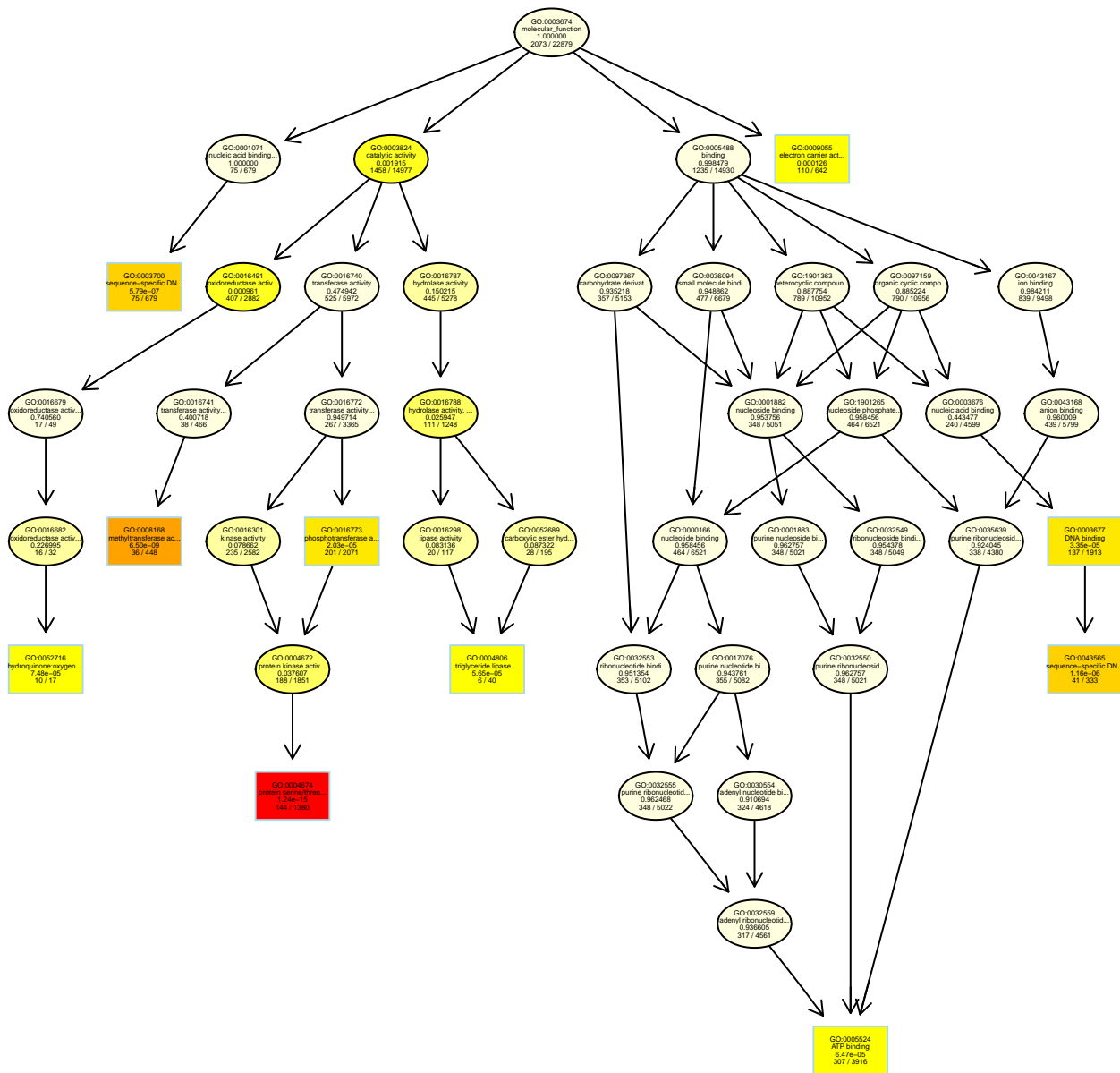

Figure S5C. GO enrichment analysis in MF of young and senescent groups

Supplement: Supplemental Information 1 [file peerj-08-8426-s001.zip › peerj-37563-supplemental_data/supplemental data/figure S1-S7/Figure S5C.pdf]

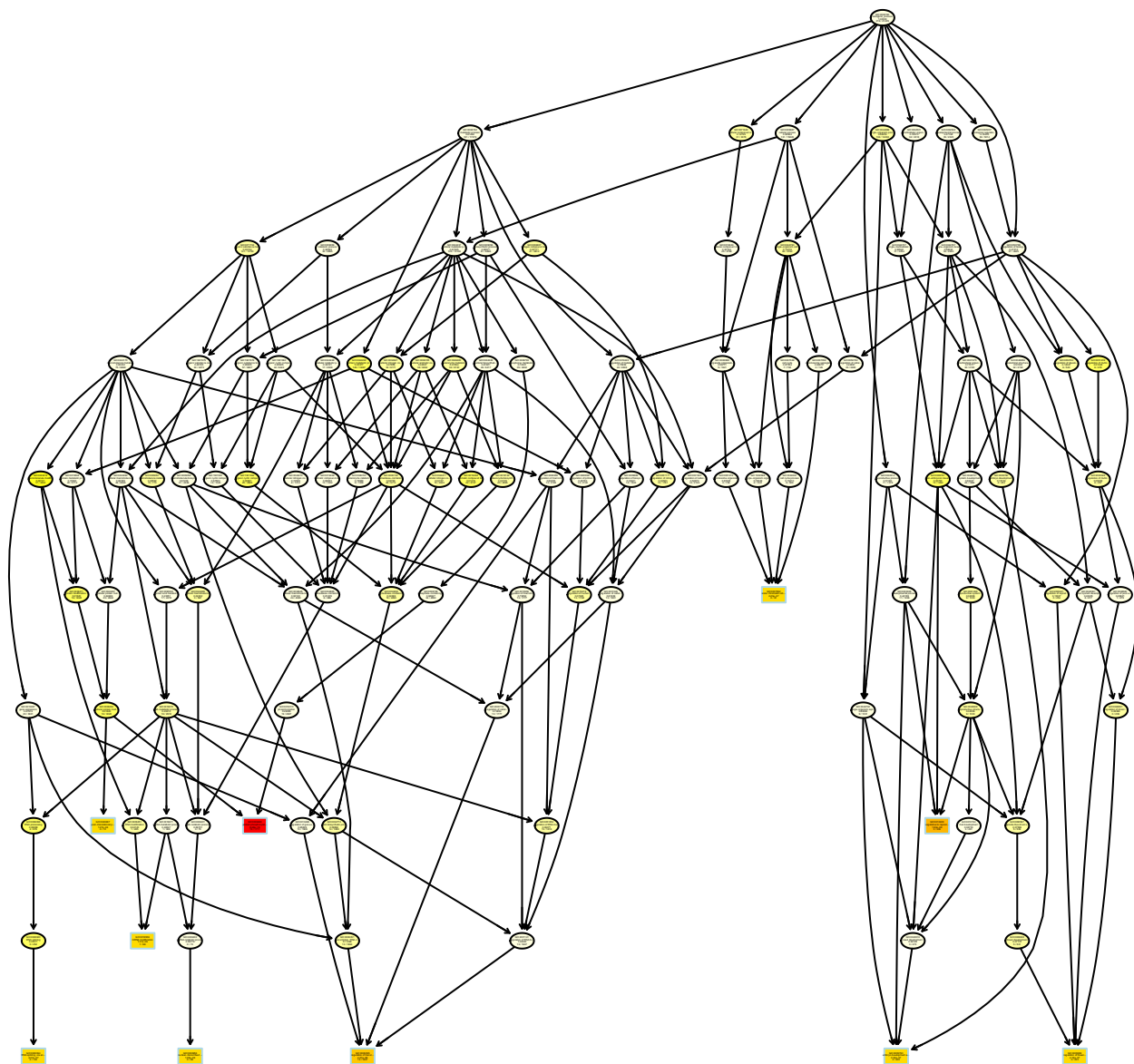

Figure S6A GO enrichment analysis in BP of mature and young groups

Supplement: Supplemental Information 1 [file peerj-08-8426-s001.zip › peerj-37563-supplemental_data/supplemental data/figure S1-S7/Figure S6A.pdf]

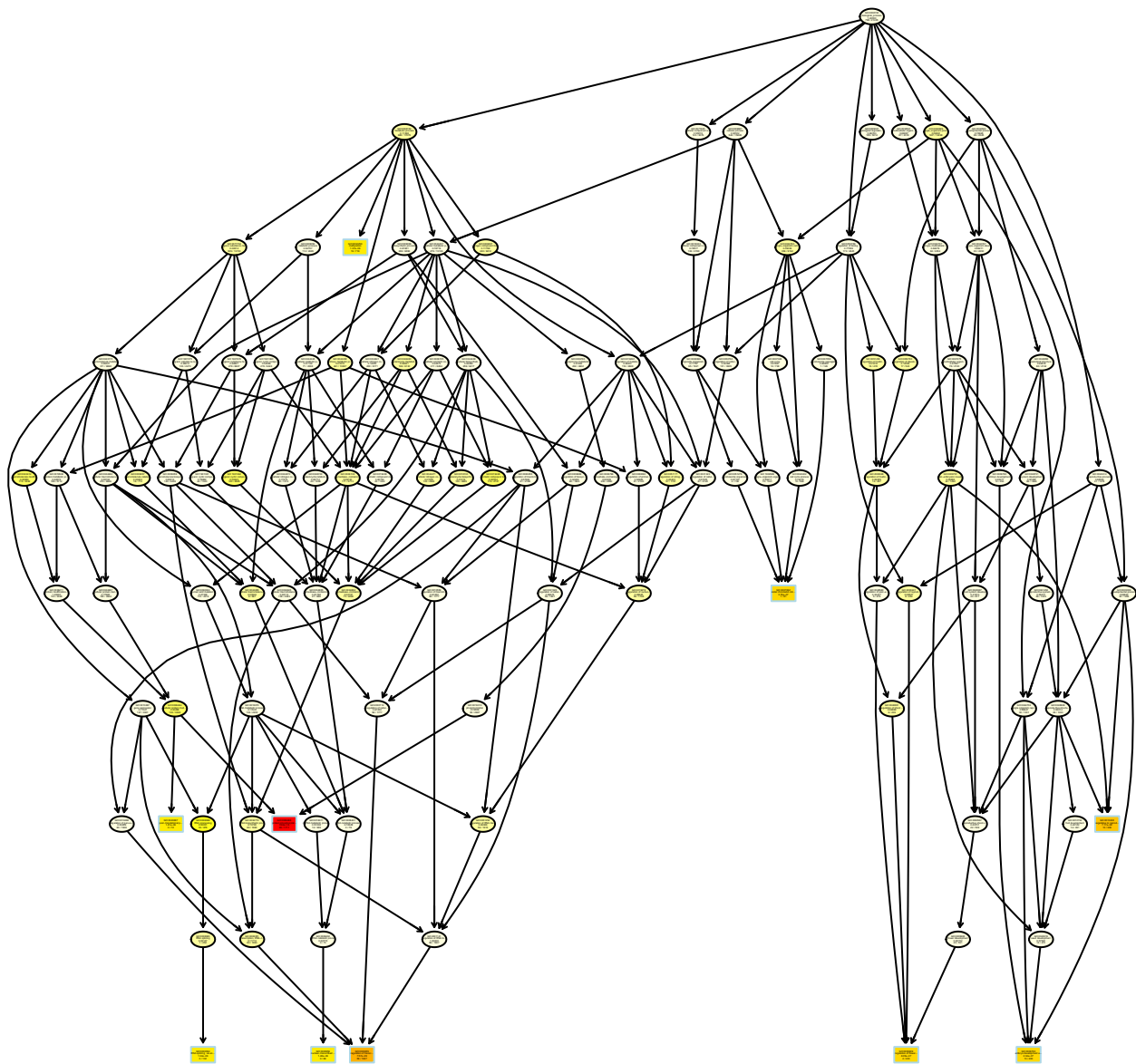

Figure S7A GO enrichment analysis in BP of mature and senescent groups

Supplement: Supplemental Information 1 [file peerj-08-8426-s001.zip › peerj-37563-supplemental_data/supplemental data/figure S1-S7/Figure S7A.pdf]
