# Supplementary material for: Global transcriptome analysis of alfalfa reveals six key biological processes of senescent leaves
Source: PeerJ. 2020 Jan 21;8:e8426. doi: 10.7717/peerj.8426 (PMC6979412; doi:10.7717/peerj.8426)
Supplement: Supplemental Information 1 [file Figure S6C.pdf › peerj-37563-supplemental_data/supplemental data/figure S1-S7/Figure S6C.pdf]

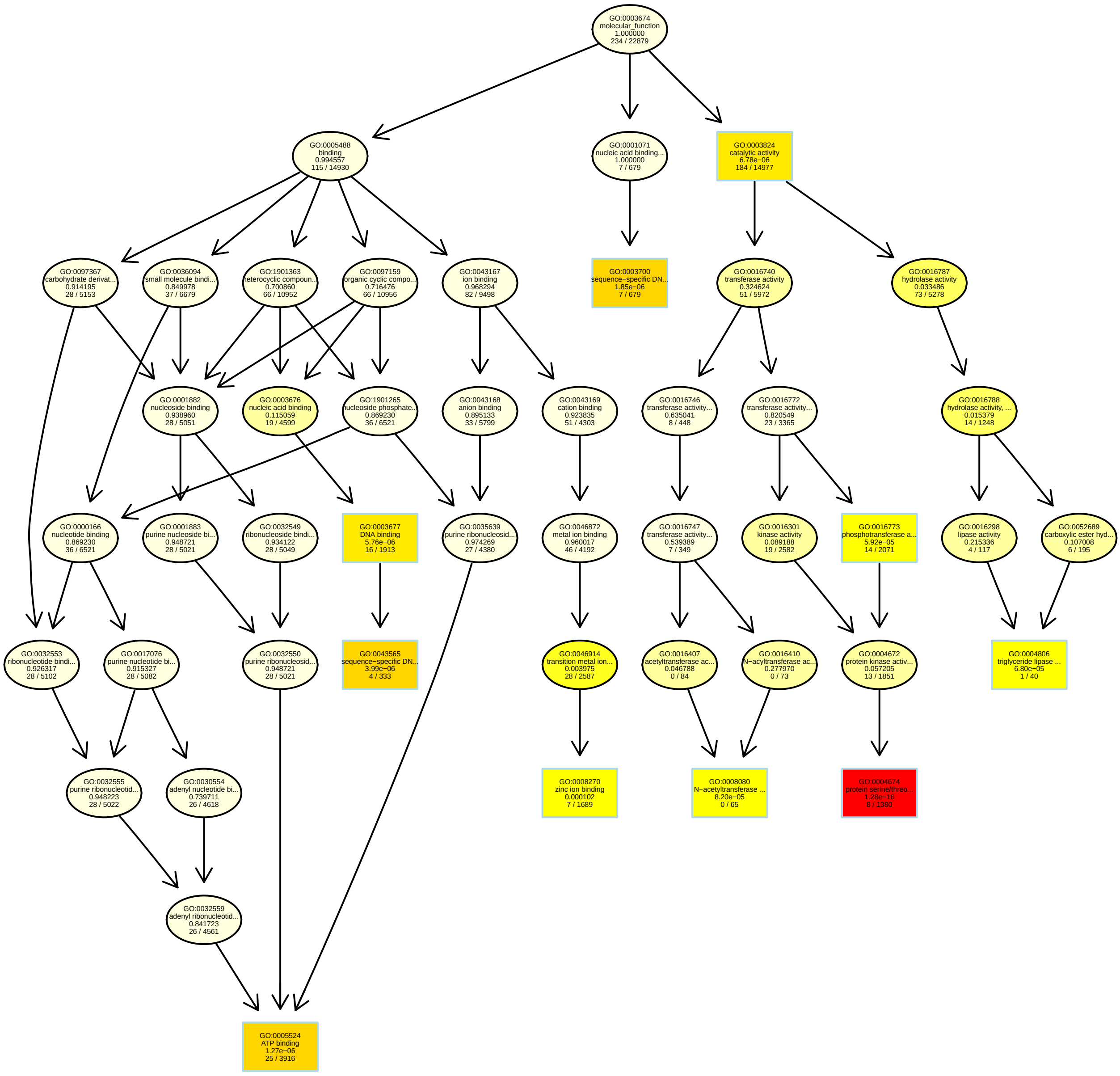

Figure S6C GO enrichment analysis in MF of mature and young groups
