# Supplementary material for: Global transcriptome analysis of alfalfa reveals six key biological processes of senescent leaves
Source: PeerJ. 2020 Jan 21;8:e8426. doi: 10.7717/peerj.8426 (PMC6979412; doi:10.7717/peerj.8426)
Supplement: Supplemental Information 1 [file peerj-08-8426-s001.zip › peerj-37563-supplemental_data/supplemental data/Table S1-S11/Table S2.doc]

**Table 2** Unigene annotation through against various databases

| Annotated in database | Number of unigene | 300≤length＜1000 | length≥1000 |
| --- | --- | --- | --- |
| Annotated in COG | 12,494 | 4,055 | 5,787 |
| Annotated in GO | 26,741 | 10,408 | 11,621 |
| Annotated in KEGG | 14,550 | 5,805 | 5,997 |
| Annotated in KOG | 22,270 | 8,235 | 9,783 |
| Annotated in Pfam | 26,517 | 9,269 | 13,108 |
| Annotated in Swissprot | 23,959 | 9,172 | 11,355 |
| Annotated in eggNOG | 36,830 | 13,799 | 15,478 |
| Annotated in Nr | 41,223 | 16,742 | 1,6731 |
| All Annotation | 43,691 | 17,353 | 16,808 |
